# Beyond vein diameter: pain phenotyping complements updated diagnostic criteria for female pelvic venous disorders

**DOI:** 10.1186/s42155-026-00764-x

**Published:** 2026-09-02

**Authors:** Vinicius Adami Vayego Fornazari, Renato Abu Hana, Grit Armstrong Adler, Sara Lojo-Lendoiro, Nucelio Lemos, Gregory Kirk Lewis, Jean-Pierre Pelage, Gloria Maria Salazar

**Affiliations:** 1 Division of Vascular and Interventional Radiology, Department of Radiology, University of Florida, College of Medicine – Jacksonville, Jacksonville, USA; 2 Complexo hospitalario de Ferrol Interventional Radiologist, Radiology Department, Hospital Arquitecto Marcide, Av. da Residencia, S/N, Ferrol, A Coruña, 15405 Spain; 3 Department of Obstetrics and Gynecology of Mount Sinai Hospital and Women’s College Hospital, University of Toronto, Toronto, Canada; 4 Division of Minimally Invasive Gynecologic Surgery, Department of Obstetrics and Gynecology, University of Florida College of Medicine - Jacksonville, Jacksonville, FL USA; 5 Department of Diagnostic Radiology, McGill University Health Centre, Royal Victoria Hospital, Montreal, QC Canada; 6 Division of Vascular Interventional Radiology, Department of Radiology, University of North Carolina, Chapel Hill, NC USA

Dear Editor,

We read with great interest the recent case–control study by Gonzales et al. demonstrating that gonadal vein (GV) diameter does not differentiate symptomatic from asymptomatic women and loses diagnostic value after adjustment for proximal venous outflow obstruction (VOO) and superficial varicosities [1]. Their findings represent more than a refinement of imaging criteria; they challenge the traditional assumption that pelvic venous disorders can be diagnosed primarily from venography or cross-sectional imaging. We believe this paradigm shift carries an important clinical implication for interventional radiologists (IRs): before venous anatomy is interpreted, the patient's pain phenotype must first be established.

Chronic pelvic pain (CPP) is recognized by the International Association for the Study of Pain as a multifactorial condition in which visceral, nociceptive, neuropathic, and centrally sensitized mechanisms frequently coexist (Fig. 1) [2, 3]. Consequently, dilated ovarian or pelvic veins should not automatically be regarded as the cause of symptoms. Similar to incidental lumbar degenerative changes in patients with back pain, pelvic varices may represent coincidental findings unless they are concordant with a clinically compatible visceral pain phenotype.

**Fig. 1:**
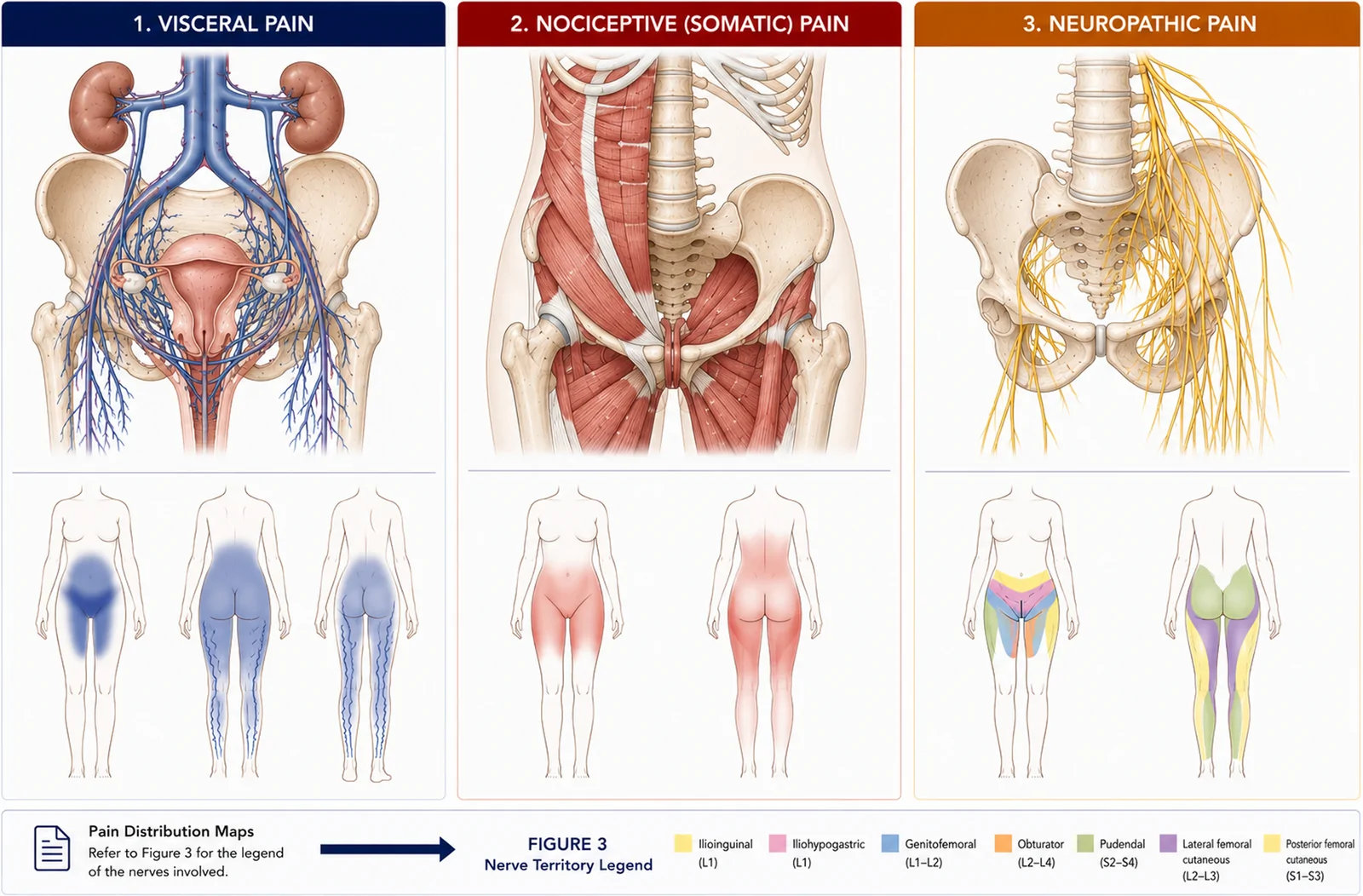
Anatomic and clinical overview of visceral, nociceptive, and neuropathic pelvic pain in women, illustrating the pelvic venous plexus and outflow pathways relevant to pelvic venous congestion syndrome, musculoskeletal/myofascial pain generators, and the pudendal/ilioinguinal nerve distributions. LE lower-extremity, VOO venous outflow obstruction

We therefore propose a practical phenotype-based triage framework (Fig. 2) and a concise differential table (Table 1) to complement—not replace—the updated diagnostic criteria proposed by Gonzales et al. [1]. Rather than asking whether pelvic veins are dilated, clinicians should first determine whether the patient’s symptoms are predominantly visceral, nociceptive/somatic, or neuropathic.

**Fig. 2:**
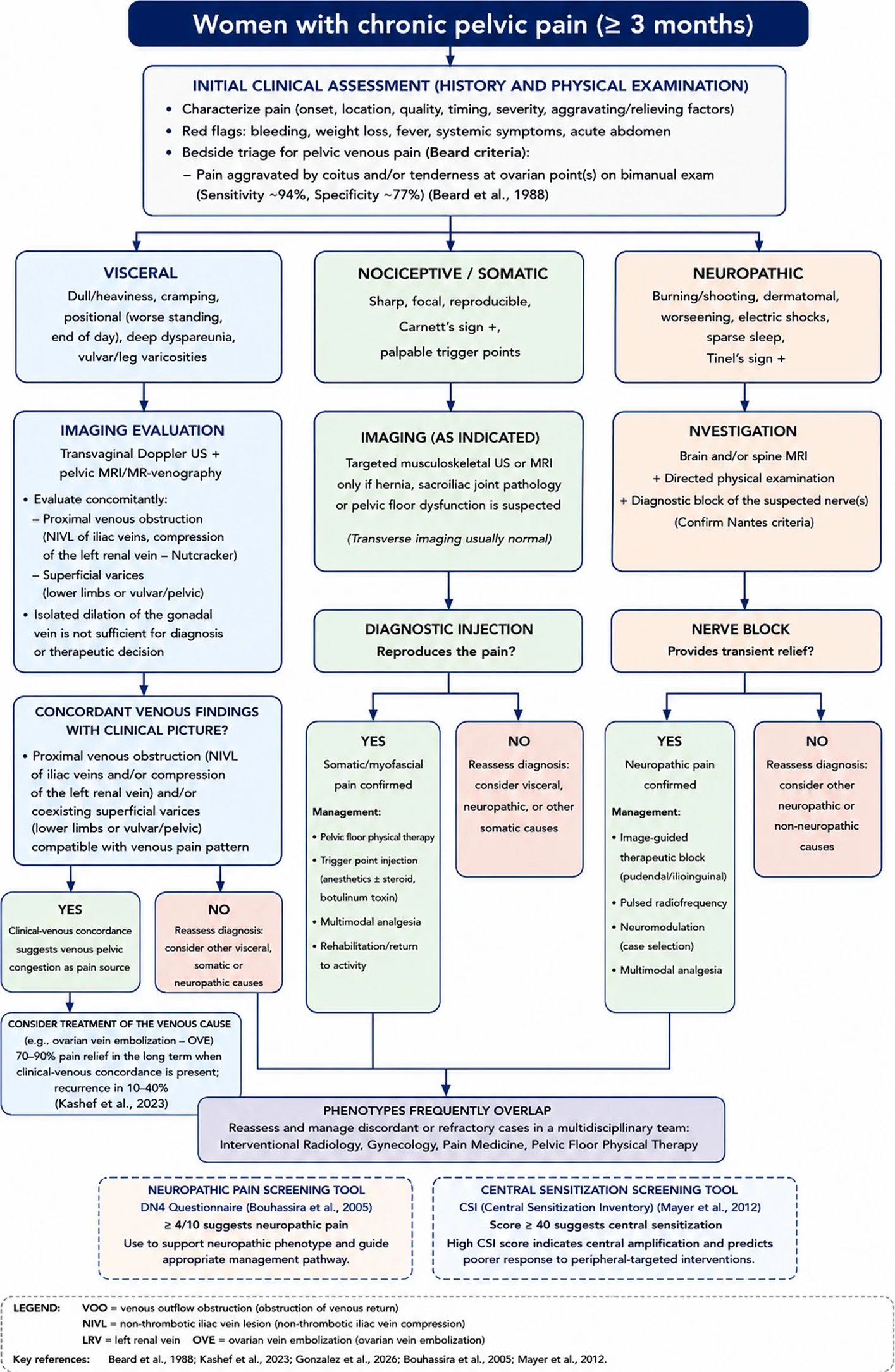
Diagnostic triage flowchart for chronic female pelvic pain in the interventional radiology setting. IR interventional radiology, MSK musculoskeletal, MRI magnetic resonance imaging, SI sacroiliac, VOO venous outflow obstruction, NIVL nonthrombotic iliac vein lesion, LRV left renal vein, LE lower-extremity, OVE ovarian/pelvic vein embolization. Phenotypes frequently overlap; discordant or refractory cases should be reassessed within a multidisciplinary chronic pelvic pain team

Patients with visceral pain typically present with poorly localized pelvic heaviness, post-coital ache, positional worsening, and a cyclical component where the pain starts at ovulation and gets progressively worse in the second phase of the menstrual cycle, with the worst days being the two just before the menstrual period. This presentation becomes even more suggestive of Pelvic Congestion Syndrome when associated vulvar or lower-extremity varicosities. In this subgroup, venous imaging becomes clinically meaningful. However, isolated GV diameter should no longer be considered sufficient evidence of symptomatic pelvic venous disease. Imaging should instead be interpreted together with proximal VOO, superficial varicosities, and the SVP classification [4].

**Table 1:** Differential features of visceral, nociceptive, and neuropathic pelvic pain in women

**Feature**	**Visceral pain** *(e.g., PVCS, endometriosis, adenomyosis)*	**Nociceptive / somatic pain** *(e.g., myofascial pelvic floor pain, abdominal wall pain)*	**Neuropathic pain** *(e.g., pudendal, ilioinguinal, iliohypogastric neuralgia)*
Pain characteristics	Dull, cramping, aching, “heaviness” or pressure; poorly localized; often bilateral	Sharp, well-defined, reproducible with palpation or movement; usually unilateral and point-tender	Burning, shooting, electric; allodynia/hyperalgesia; paresthesias
Most frequent location / referral pattern	Lower abdomen, suprapubic and adnexal (“ovarian point”); refers to low back, medial thigh, vulva; left ≥ right (left ovarian vein drains at a right angle into left renal vein)	Abdominal wall (rectus, oblique), pelvic floor/levator ani, sacroiliac joint, pubic symphysis, hip; sharply localized — patient points with one finger	Pudendal territory (perineum, vulva, anorectum); ilioinguinal/iliohypogastric/genitofemoral territory (groin, mons pubis, labia majora); dermatomal distribution
Temporal pattern	Cyclical with menses; worsens with prolonged standing and toward end of day; improves supine; postcoital ache	Positional/activity-related; reproducible with specific movement or sustained posture; constant baseline with mechanical flares	Worse with sitting; classically does not wake the patient at night (Nantes criteria); progressive, with intermittent paroxysms
Associated symptoms	Deep dyspareunia, dysmenorrhea, bloating, urinary urgency/frequency, vulvar and lower-limb varicosities	Localized muscle spasm/guarding, restricted range of motion, superficial dyspareunia if pelvic floor involved	Foreign-body/fullness sensation in vagina or rectum, superficial dyspareunia, bladder and bowel dysfunction, sexual dysfunction
Physical examination	Post-coital ache and/or deep adnexal (“ovarian point”) tenderness — 94% sensitivity, 77% specificity for PVCS [2]; pain reproduced/worsened on standing or Valsalva; visible vulvar/perineal/thigh varicosities	Positive Carnett’s sign, palpable myofascial trigger points (“jump sign”), tender levator ani on vaginal exam, positive hip/SI provocative tests (FABER, FADIR)	Positive Tinel’s sign over ischial spine/Alcock’s canal, dermatomal sensory changes on pinprick testing, tenderness along nerve course
Validated diagnostic tools	Beard clinical criteria (post-coital ache/ovarian point tenderness) [2]; Symptoms Varices Pathophysiology classification [2]; International Pelvic Pain Society history/exam form; visual analog scale, McGill Pain Questionnaire	Carnett’s sign (bedside test); diagnostic trigger-point injection with local anesthetic; digital pelvic floor tone/tenderness scoring	Nantes criteria (5 criteria) for pudendal neuralgia [4]; DN4 neuropathic pain questionnaire; diagnostic anesthetic nerve block
First-line imaging	Transvaginal Doppler ultrasound and pelvic MRI/MR-venography; isolated GV diameter thresholds (≥ 6 mm reflux, or Coakley criteria) are poorly discriminating [1] — assess concurrently for proximal VOO (nonthrombotic iliac lesion, aortomesenteric left renal vein/“nutcracker” compression) and superficial (LE/vulvar) varicosities, the stronger predictors of symptomatic disease [1]; catheter venography remains the reference standard, also therapeutic	Cross-sectional imaging usually normal/nonspecific; musculoskeletal ultrasound for trigger points or hernia; MRI for sacroiliac joint, symphysis, or hip pathology	MR neurography (nerve caliber, T2 signal, entrapment site); high-resolution pelvic MRI
Expected response to ovarian/pelvic vein embolization (OVE)	Good to excellent (70–90% long-term relief) when proximal VOO and/or superficial escape-pathway varicosities corroborate the clinical picture — not when based on GV diameter alone [1, 2]; recurrence in 10–40%	Poor / none — OVE not indicated; treating incidental venous dilation does not relieve musculoskeletal pain	None — OVE not indicated; image-guided perineural nerve block is the analogous diagnostic and therapeutic IR tool
Treatment relevant to IR	Ovarian/internal iliac vein embolization (coils ± plug or sclerosant); adjunct hormonal suppression, NSAIDs	Referral for pelvic floor physical therapy, trigger-point injection, botulinum toxin, musculoskeletal/pain medicine	Image-guided pudendal or ilioinguinal nerve block, pulsed radiofrequency, neuromodulation; surgical decompression and gabapentinoids as adjuncts

Conversely, nociceptive pain is characterized by focal tenderness, positive Carnett's sign, and myofascial trigger points. Neuropathic pain follows a dermatomal distribution and may fulfill the Nantes criteria. Validated screening tools including the DN4 questionnaire and the Central Sensitization Inventory (CSI) help identify neuropathic and centrally sensitized pain mechanisms unlikely to respond to ovarian vein embolization [3, 5, 6].

Importantly, our framework proposes a change in diagnostic sequence rather than new imaging criteria. Venous imaging should no longer be interpreted in isolation but as the final step of a structured clinical reasoning process that begins with pain phenotyping and proceeds to confirm clinical–imaging concordance. This approach may reduce the chances of ineffective embolization by improving patient selection, reserving intervention to those in whom anatomically similar venous abnormalities produce symptoms and avoiding it in those where these changes are clinically silent.

The principal contribution of Gonzales et al. is therefore not simply demonstrating that gonadal vein diameter performs poorly as a diagnostic marker. Rather, it establishes that the diagnosis of pelvic venous disorders requires integration of imaging findings with a clinically validated visceral pain phenotype. Rather than asking whether pelvic veins are dilated, interventional radiologists should first ask whether the patient's pain behaves like visceral venous pain. Only when these two dimensions are concordant should endovascular treatment be considered.

## Data Availability

All imaging and clinical information were retrospectively retrieved from the institutional PACS along with clinical observations acquired.

## References

[1] Gonzales E, Nguyen V, Risner V, du Pisanie L, Keefe N, Mody P, et al. Compression and superficial varicosities outperform gonadal vein diameter in differentiating symptomatic from asymptomatic pelvic venous disorders: a case-control study. Cardiovasc Intervent Radiol. 2026. 10.1007/s00270-026-04493-5. 42252328 PMC13337957

[2] Treede RD, Rief W, Barke A, Aziz Q, Bennett MI, Benoliel R, et al. Chronic pain as a symptom or a disease: the IASP classification of chronic pain for the International Classification of Diseases (ICD-11). Pain. 2019;160(1):19–27. 30586067 10.1097/j.pain.0000000000001384

[3] Woolf CJ. Central sensitization: implications for the diagnosis and treatment of pain. Pain. 2011;152(3 Suppl):S2-15. 10.1016/j.pain.2010.09.030 PMC3268359 20961685

[4] Kashef E, Evans E, Patel N, Agrawal D, Hemingway AP. Pelvic venous congestion syndrome: female venous congestive syndromes and endovascular treatment options. CVIR Endovasc. 2023;6:25. 10.1186/s42155-023-00365-y. 37076700 PMC10115924

[5] Bouhassira D, Attal N, Alchaar H, Boureau F, Brochet B, Bruxelle J, et al. Comparison of pain syndromes associated with nervous or somatic lesions and development of a new neuropathic pain diagnostic questionnaire (DN4). Pain. 2005;114(1–2):29–36. 15733628 10.1016/j.pain.2004.12.010

[6] Mayer TG, Neblett R, Cohen H, Howard KJ, Choi YH, Williams MJ, et al. The development and psychometric validation of the Central Sensitization Inventory. Pain Pract. 2012;12(4):276–85. 10.1111/j.1533-2500.2011.00493.x PMC3248986 21951710

